# Ecological Drivers of *Mansonella perstans* Infection in Uganda and Patterns of Co-endemicity with Lymphatic Filariasis and Malaria

**DOI:** 10.1371/journal.pntd.0004319

**Published:** 2016-01-21

**Authors:** Anna-Sofie Stensgaard, Penelope Vounatsou, Ambrose W. Onapa, Jürg Utzinger, Erling M. Pedersen, Thomas K. Kristensen, Paul E. Simonsen

**Affiliations:** 1 Center for Macroecology, Evolution and Climate, The Natural History Museum of Denmark, University of Copenhagen, Copenhagen, Denmark; 2 Department of Epidemiology and Public Health, Swiss Tropical and Public Health Institute, Basel, Switzerland; 3 University of Basel, Basel, Switzerland; 4 Envision/NTD Program, RTI, Kampala, Uganda; 5 Department of Veterinary Disease Biology, University of Copenhagen, Copenhagen, Denmark; Centers for Disease Control and Prevention, UNITED STATES

## Abstract

**Background:**

*Mansonella perstans* is a widespread, but relatively unknown human filarial parasite transmitted by *Culicoides* biting midges. Although it is found in many parts of sub-Saharan Africa, only few studies have been carried out to deepen the understanding of its ecology, epidemiology, and health consequences. Hence, knowledge about ecological drivers of the vector and parasite distribution, integral to develop spatially explicit models for disease prevention, control, and elimination strategies, is limited.

**Methodology:**

We analyzed data from a comprehensive nationwide survey of *M*. *perstans* infection conducted in 76 schools across Uganda in 2000–2003, to identify environmental drivers. A suite of Bayesian geostatistical regression models was fitted, and the best fitting model based on the deviance information criterion was utilized to predict *M*. *perstans* infection risk for all of Uganda. Additionally, we investigated co-infection rates and co-distribution with *Wuchereria bancrofti* and *Plasmodium* spp. infections observed at the same survey by mapping geographically overlapping areas.

**Principal Findings:**

Several bioclimatic factors were significantly associated with *M*. *perstans* infection levels. A spatial Bayesian regression model showed the best fit, with diurnal temperature range, normalized difference vegetation index, and cattle densities identified as significant covariates. This model was employed to predict *M*. *perstans* infection risk at non-sampled locations. The level of co-infection with *W*. *bancrofti* was low (0.3%), due to limited geographic overlap. However, where the two infections did overlap geographically, a positive association was found.

**Conclusions/Significance:**

This study presents the first geostatistical risk map for *M*. *perstans* in Uganda. We confirmed a widespread distribution of *M*. *perstans*, and identified important potential drivers of risk. The results provide new insight about the ecologic preferences of this otherwise poorly known filarial parasite and its *Culicoides* vector species in Uganda, which might be relevant for other settings in sub-Saharan Africa.

## Author Summary

*Mansonella perstans* is a widespread, but relatively unknown human filarial parasite that occurs in many parts of Africa. In a nationwide survey carried out in Uganda in 2000–2003, the distribution of *M*. *perstans* was assessed by screening school children. Here, we studied the underlying environmental drivers and ecologic correlates of the observed *M*. *perstans* prevalence patterns, produced a predictive risk map, and investigated associations with *Wuchereria bancrofti* (causative agent of lymphatic filariasis) and *Plasmodium* (causative agent of malaria). Several Bayesian geostatistical logistic regression models with and without spatially structured random effects were fitted for comparison. The model that fitted the data best was used to predict *M*. *perstans* infection risk for all of Uganda. Positive associations with *M*. *perstans* infection status were observed with cattle densities, forested areas, and vegetation greenness, whereas negative associations were observed with land surface temperature. Only a small geographic overlap was observed with *W*. *bancrofti*, and the overall level of co-infection was low (0.3%). However, where the two infections overlapped, a positive association was found. Our study presents the first nationwide geostatistical risk map for *M*. *perstans*, and gives important clues about the ecologic preferences of the still unknown main *Culicoides* vector species in Uganda.

## Introduction

The human filarial parasite *Mansonella perstans* has been considered as one of the most prevalent human parasites in Africa [1]. Despite the wide distribution, only very few studies have addressed its epidemiology and associated health consequences, and currently no effective drug therapy for treatment, control, and local elimination is available [2]. Indeed, *M*. *perstans* is viewed as one of the most neglected of the neglected tropical diseases (NTDs) [2].

On-going large-scale surveys and control programs for other filarial infections (e.g., lymphatic filariasis and onchocerciasis), considered to be of greater health importance, have largely ignored *M*. *perstans* infections, even though these filarial infections frequently co-occur. This lack of attention mainly stems from its predominance in poor rural communities, and from a paucity of a distinct and clearly recognizable clinical picture [2]. However, widespread co-occurrence with lymphatic filariasis and onchocerciasis could cause complications with regards to control program diagnosis and compliance assessment [2], and could potentially trigger adverse events during mass anti-filaricides administration [3]. It has also been suggested that there could be more subtle effects, as *M*. *perstans* might interfere with the host’s immune regulation and influence the susceptibility and effect of other, co-occurring pathogens such as *Plasmodium* spp. and HIV [2].

The geographic distribution and transmission of *M*. *perstans* is closely linked to its vectors, biting midges of the genus *Culicoides*, and their environmental requirements for breeding and feeding. *Culicoides* species are widespread throughout the world, and known to transmit a variety of pathogenic viruses, bacteria, protozoa and helminths to humans, and to domestic and wild animals [4–6]. Yet, they remain among the least studied of the Dipteran vectors [7]. As such, only a few studies have tried to incriminate the exact *Culicoides* species responsible for transmission of *M*. *perstans* in endemic areas in Africa [2]. An accurate understanding of the environmental drivers of both vector and parasite distribution is paramount for the development of spatially explicit risk models based on sound ecological principles, which can help optimize disease prevention planning, and control and elimination programs.

In 2000–2003 a national survey was conducted to map the distribution of *M*. *perstans*, concurrently with that of *Wuchereria bancrofti* [8] and *Plasmodium* parasites, in school-aged children in Uganda. While geostatistical risk and co-endemicity maps have been constructed for the two latter infections [9], *M*. *perstans* infections in Uganda have only crudely been mapped [10]. Furthermore, no risk factor analysis has been performed to identify the underlying environmental drivers of *M*. *perstans* infection, and the co-infection rates and geographic overlaps (co-distribution) between the three parasites have yet to be investigated. Delineating areas of geographic overlap, where co-infections might occur, is an important operational issue for integrated disease control planning and implementation [11].

The aim of the present study was to determine the underlying environmental drivers and ecological correlates of the observed prevalence patterns of *M*. *perstans* infection and to produce statistically robust prevalence estimates at non-sampled locations (smooth prevalence maps) across Uganda. We furthermore investigated the levels of co-infection and co-distribution with bancroftian filariasis and malaria.

## Methods

### Ethics Statement

The studies which contributed data used in this paper, received ethical clearance from the Uganda National Council for Science and Technology and were approved by the Central Scientific Ethical Committee of Denmark. Prior to each survey, meetings were held with school staff and village leaders, to explain the objectives and implications of the study. Written informed consent to participate was obtained from those examined (or from the parents/legal guardians of participants aged <15 years). At each study site, a clinical officer from a nearby health unit accompanied the team, examined all the children who were not feeling well and, if need be, either treated the children or referred them to a nearby clinic. For a full description, we refer the reader to prior publications [8, 10].

### Study Design and Parasitologic Survey Data

The surveys were carried out between October 2000 and April 2003 and included pupils aged 5–19 years from 76 Ugandan primary schools (12,207 pupils in total) covering the major topographical and ecological zones of the country (see S1 Appendix for a list of schools, with names, geographical coordinates and prevalence). Full details of the study design, data, and the procedures for selection of study sites and participants have been described elsewhere [8,10]. In brief, 100-μl blood sample was collected from each consenting child during the school day and used to prepare a thick film to examine for microfilaremia and *Plasmodium* parasites. After drying, the thick films were dehemoglobinized, fixed in methanol, stained with Giemsa, and examined under a microscope. All microfilariae observed were identified to species, using morphological criteria [12] and counted. Finger-prick samples of blood were also collected, and assayed for *W*. *bancrofti* specific circulating filarial antigens (CFA) by use of ICT cards [1]. Boys and girls were examined in approximately equal numbers.

### Environmental and Other Predictor Variables

We investigated a series of climatic and other environmental variables (Table 1) known to be of importance for the distribution of arthropod transmitted parasitic infections in the tropics, but also known ecological drivers of *Culicoides* species transmitting other parasites and viruses [7,13]. These included measures of temperature, known to influence parasite developmental rate and vectorial development rates, as well as habitat-related factors (i.e., vegetation and land use) and livestock densities that possibly influence the breeding and survival of the (unknown) *Culicoides* species believed to transmit *M*. *perstans* in Uganda.

**Table 1:** Overview, sources, and resolution of remotely sensed and other geographic information system data used for modeling.

Data type	Description	Resolution spatial (period)	Source
**Satellite imagery**			
Day-time LST	Land surface temperatures	1 x 1 km (2001–2003)	MODIS Terra^1^
Night-time LST	Land surface temperatures	1 x 1km (2001–2003)	MODIS Terra^1^
NDVI	Normalized difference vegetation index	500 x 500 m (2001–2003)	MODIS Terra^1^
Land cover	Land cover classes	1 x 1km (2002)	MODIS Terra^1^
**Other**			
Altitude	Digital elevation model derived	1 x 1 km	Shuttle Radar Topography SRTM^2^
Gridded livestock of the world (GLW2.01)	Livestock densities (head/km^2^)	1 x 1 km	FAO//GEONETWORK^3^

^1^ Moderate Resolution Imaging Spectroradiometer (MODIS); available at: https://lpdaac.usgs.gov/dataset_discovery/modis/modis_products_table (accessed: 1 October 2014).

^2^ Shuttle Radar Topography Mission (SRTM); available at: ftp://edcsgs9.cr.usgs.gov (accessed: 1 October 2014

^3^ Food and Agricultural Organization (FAO) livestock density products at GEONET; available at: http://www.fao.org/geonetwork/srv/en/main.home (accessed: 1 February 2015).

The central longitude and latitude of each school obtained using a hand-held global positioning system (GPS; Garmin eTrex, Garmin, Olathe, KS, United States of America) was utilized to define an area of 1 km radius (representing the coarsest resolution of the environmental data) encompassing the community. Average values of each environmental layer were then extracted using ArcGIS 10.1 spatial analyst extension (ESRI; Redlands, CA, United States of America). Land cover variables, calculated as the number of pixels of each category of land use, was counted within the 1 km buffer zone (using the ‘Geospatial Modeling’ environment extension [14]), and the percentage of each category calculated. For a full description of these environmental variables, see Stensgaard and colleagues [15,16].

### Statistical Analysis

Initially, a non-spatial, frequentist bivariate logistic regression analysis was conducted in Stata version 13 (Stata Corporation; College Station, TX, United States of America) to assess the relation between various environmental and habitat-related predictors of *M*. *perstans* infection status. Significant candidate factors based on likelihood ratio test (LRT) with significance levels of 5% were selected as covariates in further multivariate analyses. To avoid over-parametrization and confounding arising from correlated environmental variables within the same “environmental theme”, these were ranked by the Akaike information criterion (AIC) [17], and strongly correlated variables (Spearman rank correlation r >0.75) excluded.

Next, Bayesian multivariate non-spatial and geostatistical logistic regression models were fitted in OpenBUGS version 3.1.1. (Imperial College and Medical Research Council; London, United Kingdom) via Markov chain Monte Carlo (MCMC) methods which provide higher flexibility in fitting complex models and avoid asymptotic inference than frequentist approaches, and overcome the computational challenges encountered in likelihood-based fitting [18]. Bayesian geostatistical modeling represents the current leading edge in spatial statistics, and makes it possible to incorporate both spatial dependence and covariates, but also enables full representation of uncertainty in model outputs [19] that can be visualized, for example, as maps of prediction errors.

The association between *M*. *perstans*, *W*. *bancrofti*, and *Plasmodium* spp. was assessed using multivariate regression models on a single parasite species with all other parasite species as covariates. Demographic and cluster effects were accounted for at the unit of the school.

### Model Formulation

We assumed that the *M*. *perstans* status *Y*_*ij*_ of child *i* at location *s*_*i*_, which takes a value of 1 if the child was tested positive and 0 otherwise, follows a Bernoulli distribution *Y*_*ij*_ ~ *Ber(p*_*ij*_*)*, with *p*_*ij*_ measuring the infection risk at location *s*_*i*_. The outcome can be related to its predictors via standard multivariate logistic regression analysis. This model is given by $\text{log}it\left(p_{ij}\right)=\beta_{0}+\sum_{k=1}^{p}\;\beta_{k}X_{ij}^{\left(k\right)}+\varepsilon_{i}$ where *β*_*ij*_ = (*β*_0_, *β*_1_, *β*_2_, … *β*_*p*_) is the vector of regression coefficients and the intercept, and $X_{ij}=\left(X_{ij}^{\left(1\right)},X_{ij}^{\left(2\right)},\ldots \;X_{ij}^{p}\right)$ are the model covariates (the fixed part of the model), and *ε*_*ij*_ is a location-level exchangeable random effect that accounts for clustering of individuals in schools. They are assumed to be independent, arising from a normal distribution (∼*N*(0, *τ*^2^)) where *τ*^2^ accounts for the non-spatial variation in the infection risk data.

The spatial relationship often found among parasitemia survey locations was considered by introducing spatially correlated random effects *ϕ*_*i*_ at every sampled location *s*_*i*_, which is the standard way of incorporating geographical dependence in the model. The underlying spatial process was modeled by the residuals using the geostatistical design described in Diggle *et al*. (1998) [18] via a multivariate normal distribution, *ϕ* = (*ϕ*_1_, …. *ϕ*_*n*_)^*T*^ with variance-covariance matrix Σ. Moreover, an isotropic spatial process was assumed, i.e., Σ_*ij*_ = *σ*^2^exp(−*ρd*_*ij*_), where *d*_*ij*_ is the Euclidean distance between locations *i* and *j*, *σ*^2^ is the spatial variation (known as the sill), and *ρ* is a smoothing parameter controlling the rate of correlation decay with increasing distance. For the exponential correlation function, the minimum distance at which the spatial correlation between locations is less than 5% (range of spatial process) is calculated by 3/*ρ* for the exponential correlation structure.

To complete Bayesian model specification, independent normal prior distributions was assumed for the regression coefficients, with mean 0 and variance 100. For *σ*^2^, *τ*^2^, and *ρ* inverse gamma distributions with mean 1 and variance equal to 100 were adopted. We ran a single chain sampler with a burn-in of 5,000 iterations, followed by 100,000 iterations. Convergence was assessed by inspection of ergodic averages of selected model parameters and convergence was successfully achieved before the 100,000^th^ iteration. The strength of correlations and significance of the co-variates was assessed by inspecting the estimated odds ratios (ORs) and their Bayesian credible intervals (BCI).

For appraisal of the best fitting multivariate model, the deviance information criterion (DIC) was applied [20]. The smaller the DIC, the better the model fit. Bayesian kriging was applied to generate smooth risk maps for *M*. *perstans* prevalence based on the parameter estimates of the best fitting model [18].

## Results

### Parasitological Findings

**Fig 1:**
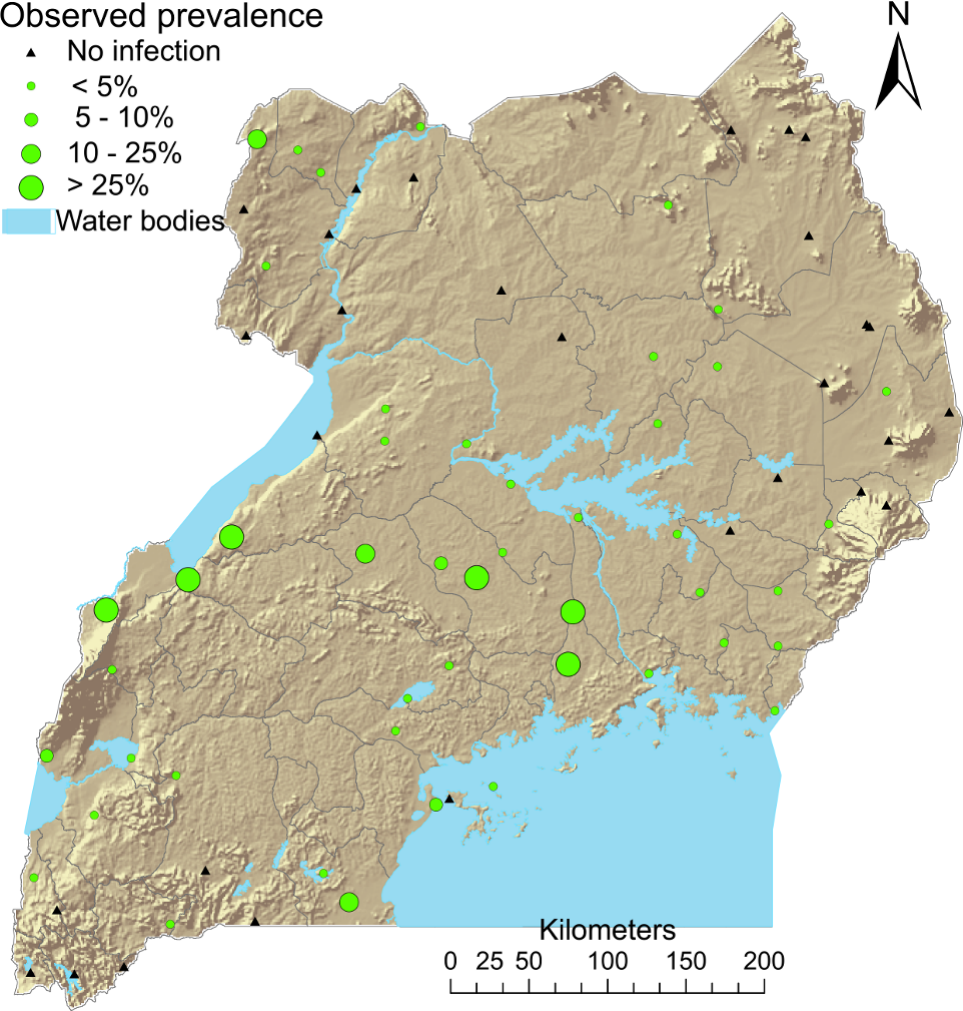
School survey locations and observed prevalence of *M*. *perstans* microfilaremia in Uganda.

Children with *M*. *perstans* microfilaremia were observed in 47 out of the 76 study sites (61.8%), with prevalence ranging from 0.4% to 72.8%. The highest prevalence was observed at sites south of Lake Albert and north-west of Lake Victoria with prevalence decreasing toward zero when moving to the north-eastern and southern sites (Fig 1).

**Fig 2:**
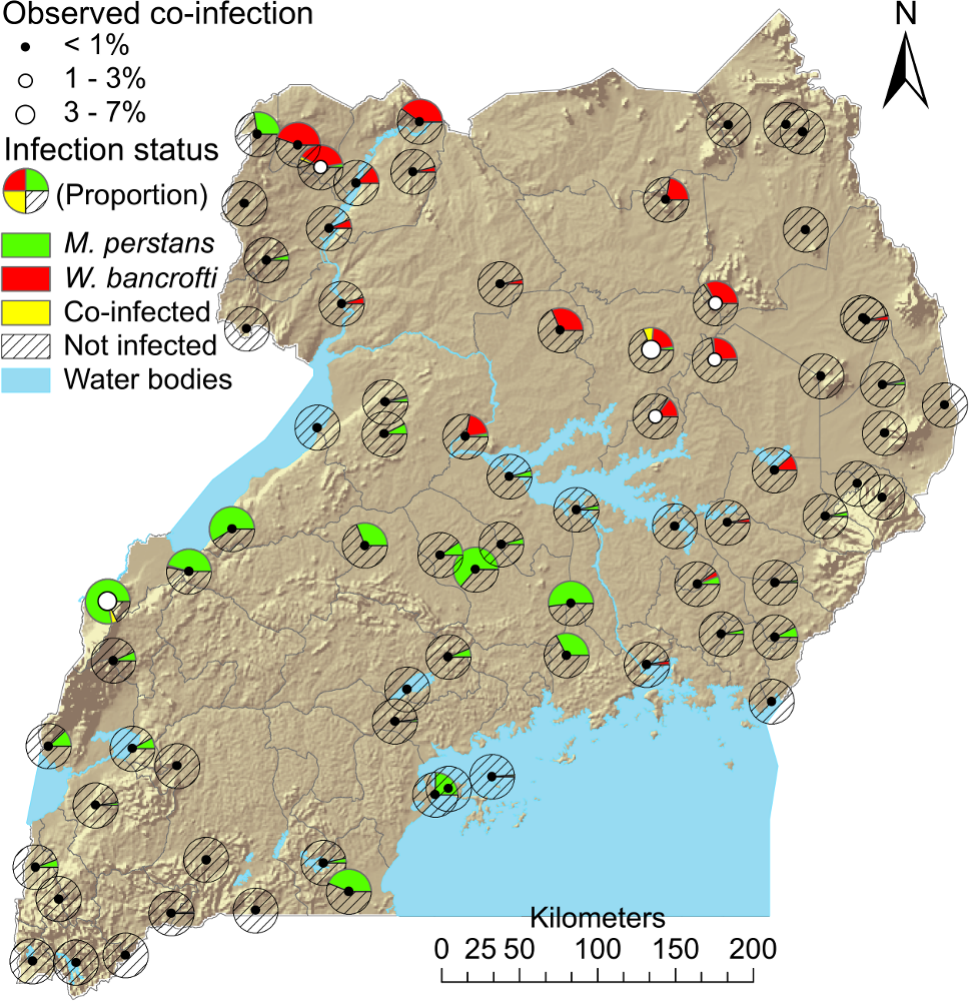
Observed proportional distribution of mono- and co-infections (yellow) with *M*. *perstans* (green) and *W*. *bancrofti* filariasis (red). Data from 11,606 pupils aged 5–19 years in 76 schools in Uganda (2000–2003).

Of the 12,207 children examined for *M*. *perstans* microfilaremia, 11,606 were examined concurrently for infection with *W*. *bancrofti* (CFA). Co-infections with *M*. *perstans* and *W*. *bancrofti* were observed in 33 individuals (0.3%) in six schools (Fig 2).

Four of these six schools were clustered together in the area north of Lake Kyoga that had relatively high *W*. *bancrofti* prevalence, but low *M*. *perstans* prevalence. However, the few children infected with *M*. *perstans* in these schools, also tested positive for *W*. *bancrofti* CFA. High levels of mono-infections with *M*. *perstans* were primarily found in the southern parts of Uganda, whereas mono-infections with *W*. *bancrofti* were restricted to the east-central northern areas of Uganda (Fig 2). Malaria has previously been found to be widespread in Uganda (see Stensgaard et al. (2011) [9] for more details).

Co-infections with *M*. *perstans* and *Plasmodium* spp were observed in 347 of 11,469 examined children (3.0%). Triple-infections with *M*. *perstans*, *Plasmodium* spp, and *W*. *bancrofti* were observed in only nine out of 11,267 (0.08%) examined children (in three schools). Co-infections and triple-infections were approximately equally distributed among age groups and sex.

### Regression Analyses

The non-spatial bivariate logistic regression analyses revealed that most of the climatic and environmental predictors were significantly associated with *M*. *perstans* prevalence (Table 2).

**Table 2:** Parameter estimates based on bivariate logistic regression models for *M*. *perstans* microfilaremia in school-aged children in Uganda (2000–2003).

Data source	Covariate	*M perstans* parasitemia		
		OR^a^	95% CI^b^	*P*-value (AIC^c^)
**School survey**	Age (5–9 years)	1.00		
	*10–14 years*	1.57	1.26, 1.96	
	*15–19 years*	2.48	1.77, 3.47	<0.001
	Sex (female)	1.00		
	*Male*	0.96	0.81, 1.14	0.660
	Season (dry)	1.00		
	*Wet*	1.52	0.39, 5.87	0.546
**Sattelite imagery**	Land surface temperature (LST)			
	*Day (wet season)*	0.79	0.66, 0.93	0.005 (3,699)
	*Day (dry season)*	0.79	0.66, 0.93	0.005 (3,699)
	*Day (annual)*	0.78	0.65, 0.91	0.003 (3,698)
	*Night (wet season)*	1.09	0.85, 1.40	0.498 (3,706)
	*Night (dry season)*	1.12	0.82, 1.52	0.473 (3,706)
	*Night (annual)*	1.10	0.81, 1.45	0.521 (3,707)
	Diurnal_diff (wet season)*	0.63	0.51, 0.78	<0.001 (3,688)
	*Diurnal_diff (dry season)*	0.65	0.53, 0.79	<0.001 (3,689)
	*Diurnal_diff (annual)*	0.63	0.51, 0.77	<0.001 (3,687)
	Normalized difference vegetation index (NDVI)			
	Wet season*	1.16	1.09, 1.23	<0.001 (3,683)
	*Dry season*	1.15	1.08, 1.21	<0.001 (3,684)
	*Annual*	1.15	1.08, 1.22	<0.001 (3,684)
	Land cover (%)			
	Forest cover*	1.06	1.02, 1.10	0.005 (3,697)
	*Open vegetation*	0.97	0.95, 0.99	0.019 (3,709)
	*Cropland*	0.98	0.95, 1.03	0.201 (3,715)
**GIS maps**	Altitude	0.99	0.99, 1.00	0.013
	Livestock			
	Cattle density*	1.04	1.02, 1.06	<0.001
	*Sheep density*	1.07	0.99, 1.16	0.105
	*Goat density*	0.99	0.98, 1.01	0.523
	*Pig density*	1.02	0.96, 1.07	0.593
	*Chicken density*	1.00	0.99, 1.00	0.673

*Chosen for further multivariate modeling. Models include a school-level random effect to account for clustering at school level.

^a^OR, odds ratios

^b^CI, 95% confidence interval

^c^AIC, Akaike information criterion.

Diurnal land surface temperature (LST) range (T_max_ minus T_min_), which was negatively associated with *M*. *perstans* prevalence, showed the best fit among the temperature variables as measured by the AIC. Among the vegetation associated variables, NDVI and the percentage of forest cover around the schools showed positive associations with *M*. *perstans* infection status, with NDVI composited over the wet season showing the best fit to the data. Among the livestock density factors, only cattle densities showed a significant (and positive) association with *M*. *perstans* infection status. Furthermore, age was a significant risk factor, with three times as high odds of being infected in the oldest age group (14–19 years) as compared to the youngest age group (5–9 years). Sex, on the other hand, was not significant at the 5% significance level and thus not included in subsequent Bayesian models.

In the Bayesian multivariate regression analyses (Table 3), the introduction of exchangeable random effects (model B), improved model performance considerably based on DIC estimates (4,991 *vs*. 3,543).

**Table 3:** Factors associated with *M*. *perstans* microfilaremia in Ugandan school-aged children based on non-spatial and spatial logistic multivariate regression modeling of national survey data (2000–2003).

Model parameter	Non-spatial(no random effect)	Non-spatial(exchangeable random effect)		Spatial model
	Model A	Model B		Model C
	OR^a^ (95% BCI^b^)	OR (95% BCI)		OR (95% BCI)
**Age (years)**				
5–9	1.00	1.00	1.00	
10–14	1.53 (1.24, 1.91)	1.59 (1.27, 1.96)	1.59 (1.27, 1.97)	
15–19	3.04 (2.23, 4.15)	2.58 (1.81, 3.54)	2.56 (1.80, 3.57)	
**Altitude**	0.99 (0.99, 1.00)	1.00 (0.99, 1.00)	1.00 (0.99, 1.00)	
**Land surface temperature (LST)**				
Diurnal range (wet season)	1.23 (1.16, 1.28)	0.72 (0.52, 0.95)	0.72 (0.50, 0.94)	
**Normalized difference vegetation index (NDVI)**				
Wet season	1.72 (1.66, 1.78)	1.07 (1.01, 1.16)	1.08 (1.02, 1.16)	
**Forest cover (%)**	0.88 (0.87, 0.90)	0.98 (0.93, 1.04)	0.98 (0.93, 1.03)	
**Livestock density**				
Cattle/km^2^	1.05 (1.04, 1.06)	1.04 (1.03, 1.06)	1.03 (1.01, 1.05)	
**Other model parameters**				
σ^2^ (random effect variance)	-	3.46 (2.00, 5.72)	3.65 (1.98, 6.71)	
ρ (rate of spatial correlation decay)	-	-	44.3 (1.49, 161.1)	
**DIC**^c^	4,991	3,543	3,389	

^a^OR, odds ratios

^b^BCI, Bayesian credible interval

^c^DIC, deviance information criterion.

The random effect had also an influence on the regression parameters of the covariates, but all covariates remained significant except forest cover. The introduction of location-specific random effect parameters into the model (model C) further increased model performance (DIC 3,543 *vs*. 3,389) suggesting that this is the best fitting model, while the covariate parameter estimates remained largely unchanged. Models 2 and 3 estimated approximately the same geographic variability *σ*^2^ (3.46 *vs*. 3.65). The estimated spatial range (above which spatial correlation drops below 5%) was 44.3, which is equivalent to about 7.5 km at the Equator.

### Risk Mapping

**Fig 3:**
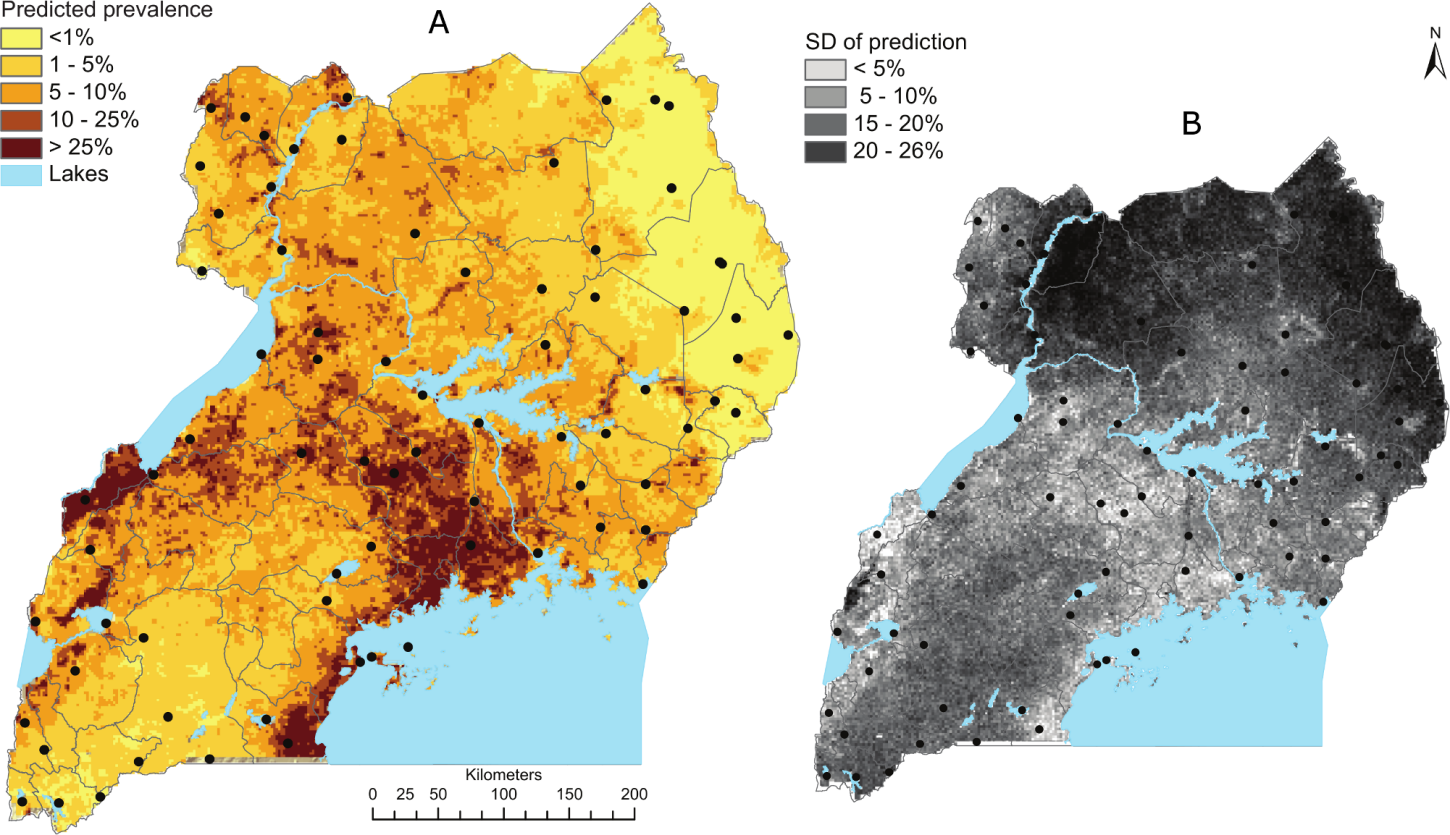
Geostatistical model-based predicted mean prevalence of *Mansonella perstans* in school-aged children in Uganda. Smooth map of the predicted mean prevalence of *M*. *perstans* (a), and the corresponding map of the standard deviations of the predictions (b), highlighting areas of high/low uncertainty associated with the model predictions.

A predictive *M*. *perstans* filariasis risk map for Uganda (Fig 3) was obtained based on the best fitting model, the spatial logistic regression model (model 3).

Highest risks were predicted in the central areas, below Lake Kyoga, with highest prevalence (>20%) south of Lake Albert, and at the northern areas and western shores of Lake Victoria. Intermediate levels (10–20%) were predicted in a belt stretching from south of Lake Albert to the north-eastern shores of Lake Victoria, but with pockets of high risk in the far north-western and south-eastern parts of the country. In contrast, low prevalence estimates (≤1%) were predominantly predicted in the north-east and central-south of Uganda. When interpreting the maps in Fig 3, it should be noted that these are based on model-predictions, and that areas with few survey points may have relatively high levels of associated prediction error. Furthermore, the predictions are based on data from school children only, and thus not necessarily representative for the adult Ugandan population infection levels, which may be considerably higher given the relationship between age and infection risk.

### Geographical Overlaps and Parasite-Parasite Associations

**Fig 4:**
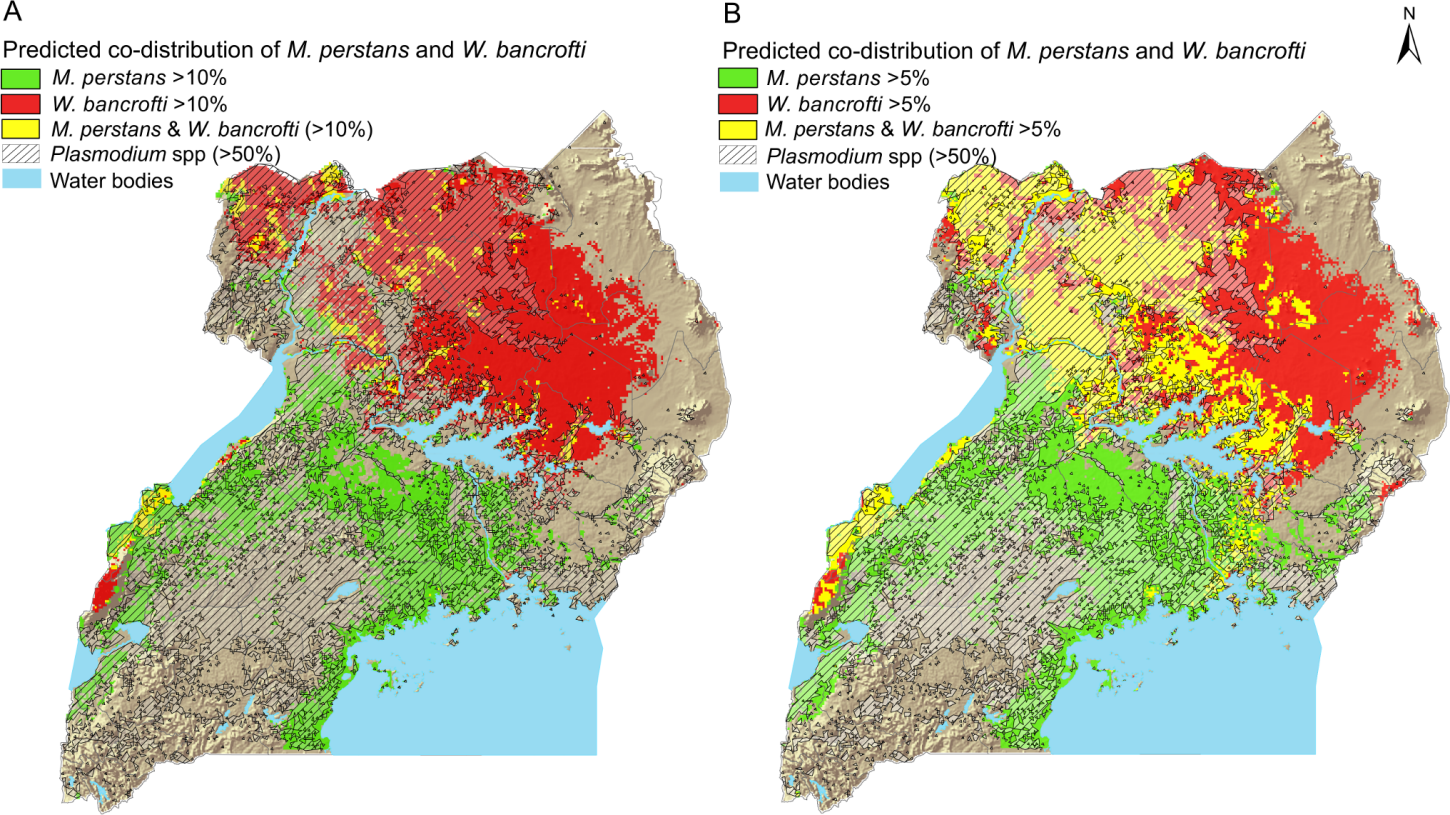
Maps of the predicted geographic co-distribution of *M*. *perstans* and *W*. *bancrofti*based on A) a 10% prevalence threshold, and B) a 5% prevalence threshold. The predicted areas are based on surveys of Ugandan school-aged children in 2000–2003 and Bayesian geostatistical model predictions of each (single) parasite infection. Predicted areas of high risk malaria (*Plasmodium* spp. infection prevalence >50%) is shown in hatch as an overlay.

The smooth map of the predicted endemic areas (predicted prevalence >5%) of *M*. *perstans* from the present study was super-imposed with a map of predicted endemic lymphatic filariasis (>5% *W*. *bancrofti* CFA prevalence) and high risk malaria (>50% *Plasmodium* spp. prevalence) previously published [8] to delineate areas of co-endemicty (Fig 4).

Results from parasite-parasite association inferred from multivariate logistic regression models revealed a significant positive association between *M*. *perstans* microfilaremia and testing positive for *W*. *bancrofti* (CFA), when clustering at the unit of schools was accounted for (Table 4). No other significant associations were found.

**Table 4:** Parasite-parasite associations as assessed by multivariate logistic regression models based on a national survey conducted in Uganda (2000–2003).

Outcome variable	Covariate	OR (95% CI)	P-value	Adjusted OR* (95% CI)	*P*-value
*M*. *perstans*	*W*. *bancrofti*	**3.26 (1.50, 6.93)**	**0.003**	**3.01 (1.43, 6.37)**	**0.005**
	*P*. *falciparum*	1.01 (0.81, 1.31)	0.983	1.19 (0.98, 1.35)	0.531
*W*. *bancrofti*	*M*. *perstans*	**3.33 (1.53, 7.14)**	**0.003**	**3.27 (1.51, 7.06)**	**0.004**
	*P*. *falciparum*	0.84 (0.62, 1.02)	0.086	0.92 (0.72, 1.10)	0.332
*P*. *falciparum*	*M*. *perstans*	1.03 (0.85, 1.21)	0.815	1.14 (0.91, 1.38)	0.524
	*W*. *bancrofti*	0.89 (0.63, 1.09)	0.092	0.90 (0.70, 1.19)	0.245

Statistically significant (p <0.05) odds ratios are highlighted in bold. OR, odds ratio; CI, confidence interval.

*Adjusted for age groups and sex.

## Discussion

The present study provided countrywide, model-based prevalence maps for *M*. *perstans* in Uganda, at a high spatial resolution. To our knowledge this is the first study to apply rigorous Bayesian geostatistical risk mapping to national survey data of this neglected human parasitic infection. The study furthermore identified risk factors and displayed high prevalence areas, and thus provides new insights into the ecological preferences of the unknown vector (*Culicoides* spp.). The resulting maps were finally combined with geostatistical risk maps previously developed for bancroftian filariasis and malaria [9], to delineate overlapping areas (co-distributions) and to investigate levels of co-infection and parasite-parasite associations. Overall, the investigations provide a deeper understanding of the zoogeographical patterns of this widespread, yet little studied parasitic infection, of importance for integrated disease control planning and implementation [11].

An increasing number of geospatial applications now analyze the relationship between parasitic infections and environmental factors, to generate predictive risk maps, including uncertainty estimates [21,22]. The majority of these studies have pertained to malaria risk [23–26], but more recently also to a number of NTDs, such as schistosomiasis [27–30], lymphatic filariasis [9], loiasis [31–32], and soil-transmitted helminthiasis [33,34]. Besides being useful for spatial targeting of control measures, surveillance, and measuring progress toward elimination, these studies can also give important new insights and clues about the ecology of the parasites and their vectors or intermediate hosts.

### Environmental Drivers

Because of the limited number of studies of *M*. *perstans* epidemiology in Africa, the current knowledge about the climatic and other environmental factors that help drive transmission of this filarial parasite is very scarce [2]. Our results indicated that high prevalence of *M*. *perstans* in Uganda was associated with cooler areas with little diurnal temperature variation, and with high NDVI values, a surrogate variable for soil moisture and correlated with vegetation biomass. A positive association was also observed with forested areas (although not significant in the final model). An association with forested ecosystems was also observed in Gabon [35,36] and Cameroon [37], and historically by Low (1903) [38], who noticed that high prevalence was associated with tropical forests alternating with swamps and other large, open ground areas. Other reports from West Africa have related the common occurrence of *M*. *perstans* along the forest fringes between the rain forest and open land, to the particular species and density of the vectors [39,40], while studies from central/southern Africa similarly indicated high prevalence in or near dense forest [41–44].

The association to forested areas, as well as banana plantations [45,46], has been linked to the importance of decomposing woody material, tree holes, and forest floor cover as breeding sites for the vector species, *Culicoides* spp. However, besides the affinity for moist substrates the some 1,400 described species of *Culicoides* show a highly diverse range of species habitat preferences, ranging from salt- and freshwater marshes, to animal dung, water logged pastures, and leaking irrigation pipes [5].

In Uganda, a total of 31 *Culicoides* species have been listed thus far [47], and several of them (e.g., *C*. *grahamii*), which have been identified as vectors of *M*. *perstans* in the Congo [48] and Cameroon [39], occur in areas predicted to be endemic for *M*. *perstans* [47]. Yet, no studies have been carried out to confirm the role of this or other *Culicoides* species in the transmission of *M*. *perstans* in Uganda.

Here, the identified environmental correlates of *M*. *perstans* give us important clues about the bionomics of the unknown vector species. Besides the climatic associations, areas of high prevalence of *M*. *perstans* were found to coincide with areas of high cattle densities (but not densities of other types of livestock). This is interesting and calls for further investigation, as *M*. *perstans* also has been shown to occur at high prevalence in Fulani nomads (cattle raising people) in northern Nigeria [49]. Possible explanations could be either a role for cattle in providing a steady source of blood meals for an opportunistic biting, or in creating habitats for larval development. Animal dung has been shown to provide important larval habitats for several *Culicoides* species [50,51] and, *C*. *grahamii*, for example, has been incriminated in blue tongue virus transmission among cattle in Kenya [52].

### Parasite Co-Distributions and Associations

While models and maps of individual parasite infections are valuable, the distribution of these infections rarely occurs independently of each other. Multiple species are often found within populations (co-endemicity) and individuals (co-infection), and co-infections are increasingly being recognized to have important health consequences [53–56]. Concomitant infections with helminths have, for example, been shown to increase susceptibility to infection with *P*. *falciparum* [57,58]. Delineating areas of geographic overlap, where co-infections might occur, is thus an important operational issue for integrated disease control planning, implementation, and evaluation.

In Uganda, several NTDs have been reported to be co-endemic [59], and also to be co-endemic with malaria [9]. Here we found that while perstans filariasis was widely overlapping with areas of high malaria risk (Fig 3), there were no significant associations between infections with *Plasmodium* spp and *M*. *perstans* and/or *W*. *bancrofti* at the individual level or at the unit of the school. A similar result was found by Nielsen et al. (2006) [60], in a study from north-eastern Tanzania, whereas Kelly-Hope et al. (2006) found a negative spatial association between *W*. *bancrofti* and *P*. *falciparum* malaria prevalence in West Africa [61].

In contrast, we observed a distinct pattern of geographic segregation between *M*. *perstans* and *W*. *bancrofti*, another filarial parasite of human health importance in Uganda (Fig 2). While *M*. *perstans* was mainly predicted in the central-to-southern parts of the country, *W*. *bancrofti* dominated in the central-northern parts. This pattern has been noted previously [10], but this is the first time a co-distribution map based on rigorous geostatistical modeling of individual infections is presented.

This very likely reflects the ecological distinctiveness of the *M*. *perstans Culicoides* spp. vector compared to that of the *Anopheles* mosquitoes transmitting *W*. *bancrofti* in Uganda. It is noted, for instance, that while high *M*. *perstans* infection risk is related to forested and densely vegetated areas, the opposite seems to apply for *W*. *bancrofti*, which showed a negative association with NDVI [9]. Similar contrasting epidemiologies have been shown for other filarial infections in Africa, i.e., between onchocerciasis and loiasis in the Democratic Republic of the Congo (DRC) [62], although *L*. *loa* and *M*. *perstans* have been found to coexist with high prevalence geographically in some African countries [35,37].

The limited geographical overlap observed in Uganda, explains the relatively low levels of overall filarial co-infection (0.8%). Yet, where the two parasites did overlap in space, in a high prevalent *W*. *bancrofti* foci in central Uganda at the northern geographical range margin of *M*. *perstans* plus a location south of Lake Albert, with high *M*. *perstans* prevalence (Figs 2 and 3), a positive association was observed between the two species. This finding warrants further investigation of potential risk factors for co-infection at the school and individual level in Uganda, and indicates that special attention should be paid to children living in geographically overlapping areas, even if these areas may be few.

In conclusion, this study adds further to our knowledge about the distinct zoogeography of filarial parasites [11] in Africa. The observed correlation between *M*. *perstans* prevalence and cattle density warrants further scientific inquiry, particularly the role played by livestock as either opportunistic blood meals (resource) for the *Culicoides* midges and/or the role of dung as larval habitats. Finally, we urge further studies based on geographically stratified field-collections of *Culicoides*, to clarify the identity, bionomics, and behavior of the vector species of *M*. *perstans* in Uganda and elsewhere in Africa, as this is a vital piece of the puzzle toward a fuller understanding of the transmission cycle and epidemiology of *M*. *perstans* infections.

## Supporting Information

S1 Appendix Table of surveyed schools with names, prevalence and geographical coordinates. (XLS)
